# Expression and Replication Studies to Identify New Candidate Genes Involved in Normal Hearing Function

**DOI:** 10.1371/journal.pone.0085352

**Published:** 2014-01-14

**Authors:** Giorgia Girotto, Dragana Vuckovic, Annalisa Buniello, Beatriz Lorente-Cánovas, Morag Lewis, Paolo Gasparini, Karen P. Steel

**Affiliations:** 1 Department of Medical Sciences, University of Trieste, Trieste, Italy; 2 Wellcome Trust Sanger Institute, Hinxton, Cambridge, United Kingdom; 3 Wolfson Centre for Age-Related Diseases, King’s College, London, United Kingdom; 4 Institute for Maternal and Child Health - IRCCS “Burlo Garofolo”, Trieste, Italy; National Cancer Institute, National Institutes of Health, United States of America

## Abstract

Considerable progress has been made in identifying deafness genes, but still little is known about the genetic basis of normal variation in hearing function. We recently carried out a Genome Wide Association Study (GWAS) of quantitative hearing traits in southern European populations and found several SNPs with suggestive but none with significant association. In the current study, we followed up these SNPs to investigate which of them might show a genuine association with auditory function using alternative approaches. Firstly, we generated a shortlist of 19 genes from the published GWAS results. Secondly, we carried out immunocytochemistry to examine expression of these 19 genes in the mouse inner ear. Twelve of them showed distinctive cochlear expression patterns. Four showed expression restricted to sensory hair cells (Csmd1, Arsg, Slc16a6 and Gabrg3), one only in marginal cells of the stria vascularis (Dclk1) while the others (Ptprd, Grm8, GlyBP, Evi5, Rimbp2, Ank2, Cdh13) in multiple cochlear cell types. In the third step, we tested these 12 genes for replication of association in an independent set of samples from the Caucasus and Central Asia. Nine out of them showed nominally significant association (p<0.05). In particular, 4 were replicated at the same SNP and with the same effect direction while the remaining 5 showed a significant association in a gene-based test. Finally, to look for genotype-phenotype relationship, the audiometric profiles of the three genotypes of the most strongly associated gene variants were analyzed. Seven out of the 9 replicated genes (*CDH13, GRM8, ANK2, SLC16A6, ARSG, RIMBP2* and *DCLK1*) showed an audiometric pattern with differences between different genotypes further supporting their role in hearing function. These data demonstrate the usefulness of this multistep approach in providing new insights into the molecular basis of hearing and may suggest new targets for treatment and prevention of hearing impairment.

## Introduction

The auditory system is one of the most complex senses in humans. The mammalian inner ear is an intricate structure functionally organized into auditory and vestibular components designed to transform mechanical energy into electrical stimuli, which will eventually be translated in the brain [1]. The hearing system is characterized by three structures: a) the outer ear, b) the middle ear, and c) the cochlea of the inner ear, that all play a role in hearing function. The cochlea contains sensory hair cells (HC), mechanoreceptors that initiate action potentials in response to sound, as well as surrounding supporting cells. All of these highly differentiated cells perform specialized functions, requiring the expression of unique combinations of genes [2].

Given the complexity of the hearing mechanism, it should come as no surprise that many genes are involved in hearing. So far, more than 140 loci associated with non-syndromic hearing loss have been mapped, and approximately 65 genes identified in humans (http://hereditaryhearingloss.org/). In mouse models more than 230 genes have been so far described (http://hearingimpairment.jax.org/index.html) to cause inner ear malformations or dysfunction. Despite the identification of these genes involved in deafness, the molecular basis of variation of normal hearing function is still largely unknown. We recently reported results from a genome-wide association study (GWAS) on normal hearing function in humans, giving an insight into some candidate genes that may be involved in the trait [3]. Several audiometric measurements were tested as quantitative traits and hundreds of SNPs had suggestive p-values (∼10^−5^ or less). Here we report our follow-up investigations of the SNPs with suggestive p values found by the previous GWAS [3]. The approach is based on four sequential steps: a) selection of a short list of candidate genes from the previous study, b) evaluation of their expression patterns in the mouse inner ear, c) genetic association replication of those genes clearly expressed in the cochlea in a new independent cohort from the Silk Road, d) examination of genotype/phenotype relationships for each of the replicated genes or SNPs.

## Materials and Methods

### Ethics Statement

Human. Consent forms for clinical and genetic studies were signed by each participant and all research was conducted according to the ethical standards defined by the Helsinki declaration. The study was approved by the Institutional Review Board of IRCCS Burlo Garofolo PROT CE/v-78.

Mice. Mouse studies were carried out in accordance with UK Home Office regulations and the UK Animals (Scientific Procedures) Act of 1986 (ASPA) under a UK Home Office licence, and the study was approved by the Wellcome Trust Sanger Institute’s Ethical Review Committee. Mice were culled using methods approved under this licence to minimize any possibility of suffering.

### Sample Collection, Preparation and Genotyping of the Replication Cohort

We collected samples from 493 people aged >18 years old from several rural communities located along the Silk Road during the Marco Polo Scientific Expedition [4] (see Figure S1). Saliva samples were collected using the Oragene kit (DNA Genotek Inc.) and DNA extracted according to supplier’s protocols. DNA samples were genotyped with the Illumina 700K platform. In order to perform a consistent replication study, the collection of genotype and phenotype data followed the same protocols as reported for the previously published work [3]. After standard quality control, all genotypes were checked to ensure that they were reported with the coordinates of the 1000 Genome Project (releases build 37) reference data and on the forward strand of the reference genome [5].

### Imputation

Genotype imputation was conducted using SHAPEIT2 [6] for the phasing step and IMPUTE2 [7] for the imputation to the 1000 Genomes phase I v3 reference set [5]. After imputation SNPs with minor allele frequency (MAF) <0.01 or imputation quality score (Info) <0.4 were excluded from the statistical analyses.

### Audiometric Evaluation

Audiometric tests and a careful clinical examination (i.e. psychological, neurological, cardiological, etc.) were carried out for each individual of the Silk Road cohort. Thresholds for different frequencies (0.25, 0.5, 1, 2, 4, 6, 8 kHz) were measured and the pure-tone averages of air-conduction thresholds (PTA at the lower 0.25, 0.5 and 1 kHz, middle 0.5, 1 and 2 kHz, and high frequencies 4, 8 kHz) were calculated. Furthermore the first three Principal Components (PC1, PC2 and PC3) for all the frequencies combined were computed in order to summarize the audiometric data. Single frequencies, PTAs and PCs were used to run the association studies. Although many different measurements were collected and analyzed, they were not independant; in fact PC1 alone accounted for approximately 80% of the total variance so we are not analyzing multiple phenotypes. Familial forms of inherited hearing loss as well as subjects affected by diabetes or other systemic disorders facilitating the development of hearing loss were excluded from the study.

### Selection of Candidate Genes

From our earlier GWAS analysis, we started with a list of 683 SNPs that were associated with the hearing trait with a p value of 10^−5^ or lower. This number of SNPs was reduced to 84 by a) examining a region 250 kb upstream and downstream of each SNP and selecting the most significantly-associated SNP, removing the less significant SNPs in the region; b) removing SNPs that marked gene deserts, where there was no annotated gene in the region +/−250 kb; c) removing SNPs that marked only genes with unknown functions such as *LOC* or *FAM* designations; and d) removing SNPs that marked genes already known to be involved in diseases unrelated to hearing function. The 84 SNPs were then reduced to a total of 19 genes prioritized by the availability of antibodies for expression studies plus at least one of the following criteria a) the lowest p values of 10^−7^; b) genes belonging to gene families previously associated with hereditary hearing loss; c) expression in the inner ear from genome-wide approaches reported in databases including Eurexpress, MGI and the NCBI EST list; or d) genes with potential functional links to the genes selected by the above criteria occurring in the same *in silico* pathways. For each SNP the closest gene was selected, but in some cases, more than one gene was included based on linkage to a single SNP (see Table 1).

**Table 1:** List of candidate genes derived from previously published Meta-Analysis [3] with inclusion criteria satisfied.

SNP	GENE	SNP-GENE distance OR SNPposition in the gene	Similarity withHHL genes	Sugg.p-value†	H.Sugg.p-value‡	Availability of antibodies
rs8077384	*AMZ2*	∼39 kb		X		X
rs17045059	*ANK2*	NM_001127493.1:c.−40+35627A>T		X		X
rs8077384	*ARSG*	∼92 kb		X		X
rs17195859	*CDH13*	NM_001220488.1:c.778−59471G>A	X	X		X
rs898967	*CMIP*	NM_030629.2:c.18+37681C>T		X		X
rs10091102	*CSMD1*	NM_033225.5:c.4985−1219C>G		X		X
rs9574464	*DCLK1*	∼30 kb			X	X
rs6673959	*DFFB*	∼321 kb		X		X
rs12758887	*EVI5*	∼136 kb		X		X
rs11159133	*FOS*	∼10 kb		X		X
rs7182802	*GABRG3*	NM_001270873.1:c.270+75238C>T	X	X		X
rs2687481	*GRM8*	∼210 kb	X		X	X
rs6673959	*KIAA0562*	∼350 kb		X		X
rs10815873	*PTPRD*	NM_002839.3:c.4086+398C>A	X		X	X
rs10848114	*RIMBP2*	NM_015347.4:c.−4+11838G>A		X		X
rs12758887	*RPL5*	∼136 kb		X		X
rs8077384	*SLC16A6*	∼100 kb	X	X		X
rs12758887	*SNORA66*	∼87 kb		X		in situ probes used
rs12758887	*SNORD21*	∼91 kb		X		in situ probes used

Key: Sugg. p-value† = suggestive p-value: p∼10-5,10-6; H.Sugg. p-value‡ = highly suggestive p-value: p∼10-7 [3].

### Expression Studies

Wild-type mice at postnatal day 4 or 5 (P4 and P5) from the albino C57BL/6J-*Tyr^c-Brd^* or pigmented C3HeB/FeJ inbred strains were used for all experiments. This age was selected because most genes known to be required for normal hearing are expressed by this stage but the bone is soft enough to avoid decalcification – a prolonged step that can affect antigen preservation. The heads of all samples were dissected in PBS before fixation for two days in 10% formalin at 4°C, washing, dehydrating and embedding in paraffin wax. Embedded samples were cut into 8 µm thick sections along the sagittal plane. Immunohistochemistry was then carried out on slides using the Ventana Discovery machines with the manufacturer’s reagents CC1 (cat.no 950-124), EZPrep (cat.no 950-100), LCS (cat.no 650-010), RiboWash (cat.no 760-105), Reaction Buffer (cat.no 95-300), and RiboCC (cat.no 760-107) and according to the manufacturer’s instructions. The DABMap™ Kit (Ventana; cat.no 760-124) with hematoxylin counterstain (cat.no 760-2021) and bluing reagent (cat.no 760-2037) was used. All antibodies were diluted in ‘Antibody staining solution’: 10% fetal calf serum, 0.1% Triton, 2% BSA and 0.5% sodium azide in PBS. The primary and secondary antibodies with the relevant concentrations used are reported in the Table S1. For each gene, slides covering the entire inner ear (cochlea and vestibular system) for at least three different mice were stained, and the observed expression patterns were considered reliable only if present in all three samples. Stained slides were examined and images obtained using an AxioCam HRc camera mounted on a Zeiss microscope. Images were then processed in Photoshop CS5 extended.

For immunofluorescence analysis of whole mounts, cochleae were dissected at P4–P5 and fixed in 4% paraformaldehyde for 2 hours. They were then washed in PBS, permeabilized in 1% PBS/Triton-X-100 (PBT) and blocked with 10% sheep serum. Then, they were incubated with the goat polyclonal primary antibody anti-CSMD1 (Santa Cruz, sc-68280, 1∶200) overnight at 4°C. After washes with PBT, samples were incubated with anti-goat Alexa Fluor 488 secondary antibody (Invitrogen, anti-goat, diluted 1∶300) and Rhodamine/Phalloidin (Invitrogen, diluted 1∶100). Samples were mounted in Prolong Gold Antifading reagent (Invitrogen). Images were acquired on an LSM 510 Meta confocal microscope (Zeiss, Welwyn Garden City). Post-acquisition image analyses were performed using Adobe Photoshop CS2.

### Statistical Analysis

Genes showing robust expression patterns in the cochlea were further analyzed for replication. Association analysis of GWAS-type was carried out in the Silk Road cohort using the GRAMMAR-Gamma method as implemented in the GenABEL suit [8] for genotyped SNPs and MixABEL [9] for imputed data. Data were adjusted to account for sex, age and genomic kinship as previously described [3] and different genetic models were tested (additive, recessive, dominant and overdominant). Moreover, in order to account for different population-specific effects, that are difficult to replicate at the SNP level, a gene-based test was performed as follows: 1) all the hearing traits were adjusted for covariates and genomic relatedness, using GRAMMAR-Gamma; 2) for each gene only intragenic SNPs were selected; 3) principal components (PC) of the genotypes coded as numerical values (0,1,2) were computed and only those explaining the largest amount of the genetic variance were taken into account (i.e. if N is the total number of intragenic SNPs then PCs explaining more than 1/N variance were chosen); 4) the selected PCs were used as multiple regressors for the corrected traits. This methodology was described and tested by Wang and Abbott [10]. Significance level considered was nominal significance (5%), which is usually accepted for replication studies, especially when generalizing findings to non-European populations as in our case [11]. Furthermore we did not correct for the different genetic models tested because these are not four independent tests [12].

### Genotype-phenotype Correlations

The audiometric phenotype was compared in a subset of 2904 subjects from Italy, Caucasus and Central Asia (Silk Road cohort) by focusing on the three genotypes (AA, AB and BB) of each replicated top SNP to study any differences among them. Among these SNPs, the homozygotes for the minor allele group ranged from 34 to 642 people. After computing mean values and standard errors for each frequency (0.25, 0.5, 1, 2, 4, 8 kHz) and for each genotype, results were plotted to form three different audiometric curves. For each plot, the Y-axis represents age-adjusted threshold values measured in decibels sensation level (from 50 dB SL to −10 dB SL) and the X-axis represents frequency measured in kHz. The points represent the mean values for each genotype at each frequency and standard errors of the traits are shown. The profiles were then visually inspected.

## Results

**Figure 1:**
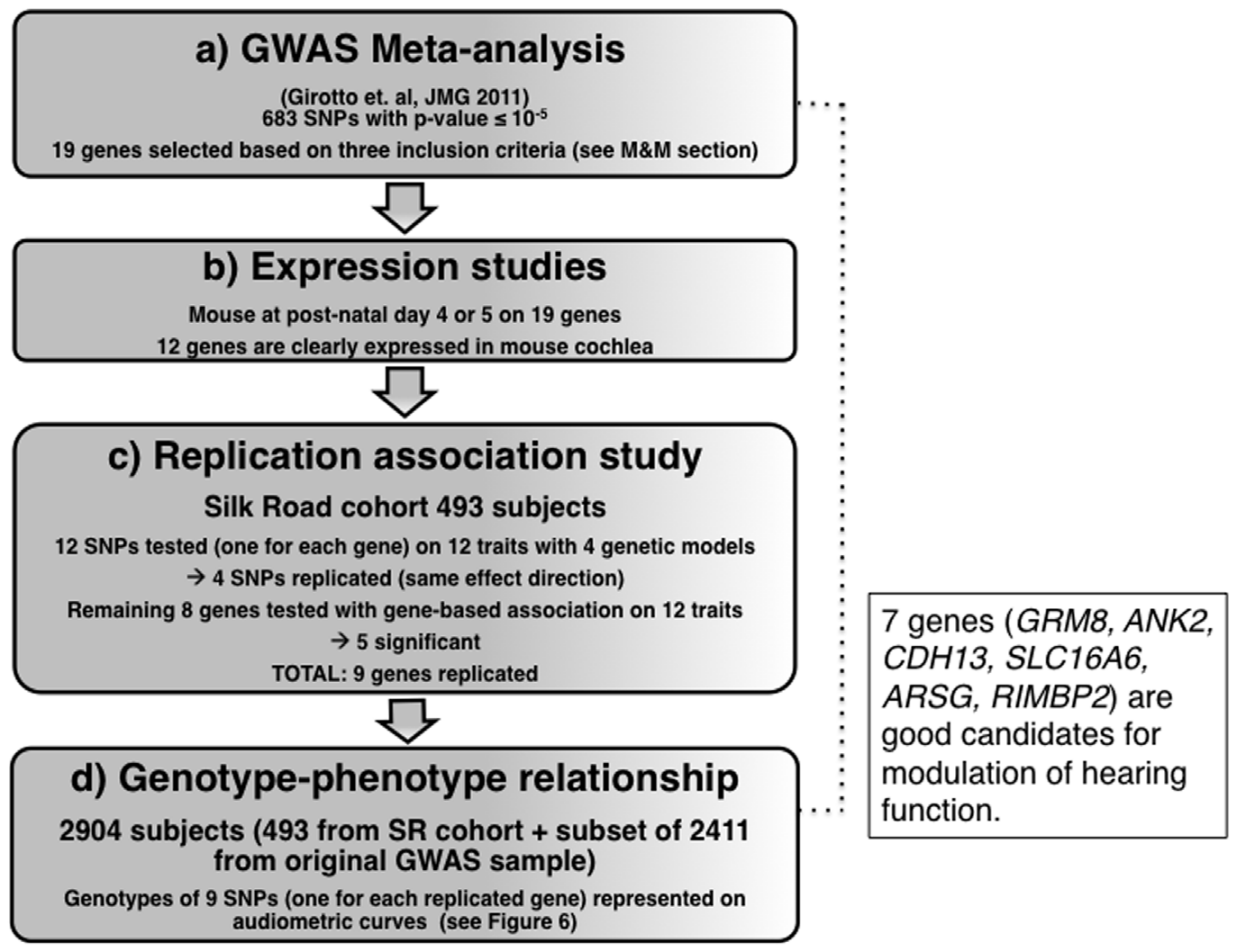
Analysis flow chart. The diagram illustrates the 4 steps defining our strategy. Relevant details for each step are given: GWAS meta-analysis description, expression studies in the mouse cochlea, replication association study in Silk Road cohort and genotype-phenotype relationship.

In this study, a list of 19 genes were selected from a list with suggestive significance recently reported in a GWAS meta-analysis of different quantitative hearing traits [3], for further investigation of their possible role in hearing function. Those with evident staining within the cochlea (expression step) underwent a replication study in an independent cohort from the Silk Road and, for the replicated ones, genotype-phenotype correlations were carried out (validation step) (Fig. 1).

The list of 19 genes was selected following the criteria described in the methods. Details of each gene are given in Tables 1 and 2, including information about expression reported in several databases and descriptions of any mouse mutant phenotypes available. In order to define their expression pattern in the mouse cochlea, which has not been reported before, immunohistochemistry studies in mice at P4–P5 were performed. Results from these studies demonstrated specific cochlear expression patterns for 12 of them (63%) at this age. All 12 genes gave the same levels of staining from the apex to the base of the cochlea. Furthermore, in 9 cases clear labelling was also evident in the vestibular system.

**Table 2:** Further details of shortlisted candidate genes.

Gene	MGI	Morton	SHIELD*	SHIELD**	DFN	Mouse knockout phenotype (MGI)
*AMZ2*	No	No	No	Yes	No	N/A
*ANK2*	No	Yes	No	No	No	Homozygous mutation of this gene results in death by postnatal day 8, although some animals survive to P20. Mutant animals display reduced body size, impaired balance and locomotion, brain structure dysmorphologies, abnormal lens, and optic nerve degeneration.
*ARSG*	No	No	No	Yes	No	Mice homozygous for a null mutation display lysosomal storage pathology in the nervous system and peripheral tissues, including the liver and kidneys, resulting in Purkinje cell loss and age dependent cognitive impairment.
*CDH13*	No	No	No	No	No	Mice homozygous for a null allele exhibit decreased retinal neovascularization and increased adiponectin levels.
*CMIP*	No	No	Yes (p<0.089)	Yes	OTSC4	N/A
*CSMD1*	No	No	Yes (p<0.076)	No	DFNM2 WS2C	No abnormal phenotype reported; Mice exhibit normal pre-pulse inhibition, social interaction, sucrose preference and d-amphetamine sensitivity.
*DCLK1*	No	No	No	Yes	No	Mice homozygous for a null allele lack the corpus callosum and hippocampal commissure and show aberrant interhemispheric axonal projections. Mice homozygous for a different null allele have normal gross brain architecture but show axonal and dendritic defects following knockdown of Dcx expression.
*DFFB*	No	No	No	Yes	No	Mice homozygous for a knock-out allele are viable, fertile and developmentally normal; however, mutant thymocytes and other cell types fail to undergo apoptotic DNA fragmentation in response to dexamethasone or other apoptotic stimuli.
*EVI5*	No	Yes	No	Yes	No	N/A
*FOS*	No	No	No	Yes	No	Diminished responses to sharp noises. Null mutants are growth-retarded, most dying perinatally. Survivors have osteopetrosis and abnormal tooth eruption, gametogenesis, hemopoiesis, behavior and photoreceptor apoptosis. Hippocampal-specific mutants have seizures and highly excitable neurons.
*GABRG3*	No	No	No	Yes	No	N/A
*GRM8*	No	No	No	No	DFNB14 DFNB17	Mice homozygous for a knock-out allele are overweight and mildly insulin resistant, and display increased anxiety-related responses and reduced exploration in a new environment. Mice homozygous for a different knock-out allele exhibit altered excitatory responses in the dentate gyrus.
*KIAA0562*	No	No	No	Yes	No	N/A
*PTPRD*	No	Yes	No	Yes	No	Homozygotes for a targeted null mutation exhibit impaired learning of spatial tasks, enhanced long-term potentiation at hippocampal synapses, and high mortality associated with reduced food intake.
*RIMBP2*	No	No	No	Yes	DFNA25 DFNA41	N/A
*RPL5*	No	Yes	No	N/A	No	N/A
*SLC16A6*	No	No	No	Yes	No	N/A
*SNORA66*	No	No	N/A	N/A	No	N/A
*SNORD21*	No	No	N/A	N/A	No	N/A

Expression in the ear reported in Mouse Genome Informatics (MGI, http://www.informatics.jax.org/); the Morton cDNA human foetal cochlea cDNA library (Morton, http://brighamandwomens.org/Research/labs/BWH_Hearing/Cochlear_ESTs.aspx); the SHIELD database (SHIELD: Shared Harvard Inner-Ear Laboratory Database, https://shield.hms.harvard.edu/); plus localisation within a reported human deafness locus and phenotypic reports from MGI of any mouse mutations of the gene.

Key: SHIELD* = SHIELD - enriched in hair cells compared to supporting cells; SHIELD** = SHIELD - auditory/vestibular ganglia; DFN = within a human deafness locus?; N/A = data not available.

A summary of the distribution of labeling is given in Table 3. According to the inner ear distribution we could divide the pattern of expression into three different categories.

**Table 3:** List of genes with specific labeling patterns in the cochlea with a summary of expression results.

Gene	ihc	ohc	rc	sp	tm	bm	sv	pc	Dc	cC	Hc	Ko	sg	maculae	cristae
Ank2								+	+		+			N/A	N/A
Arsg	+	+												+	+
Cdh13	+	+		+			+	+	+	+	+	+		N/A	N/A
Csmd1	+	+													
Dclk1							+							+	+
Evi5	+	+	+	+			+	+	+	+	+	+	+	+	+
Gabrg3	+	+													+
GlyBP	+	+		+	+	+					+			+	+
Grm8	+	+	+	+			+			+	+	+	+	+	+
Ptprd	+	+					+					+	+	+	+
Rimbp2	+	+	+	+	+		+						+	+	+
Slc16a6		+												+	+

**Key**: **ihc**, inner hair cells; **i/obc**, inner and outer border cells; **ohc**, outer hair cells; **rc**, root cells; **sp**, spiral prominence; **tm**, tectorial membrane; **bm**, basilar membrane; **sv**, stria vascularis; **pc**, pillar cells; **Dc**, Deiter cells; **cC**, cells of Claudius; **Hc**, Hensen cells; **Ko**, Kölliker’s organ; **sg**, spiral ganglion; N/A, not available.

### 1) Expression in Marginal Cells of the Stria Vascularis

**Figure 2:**
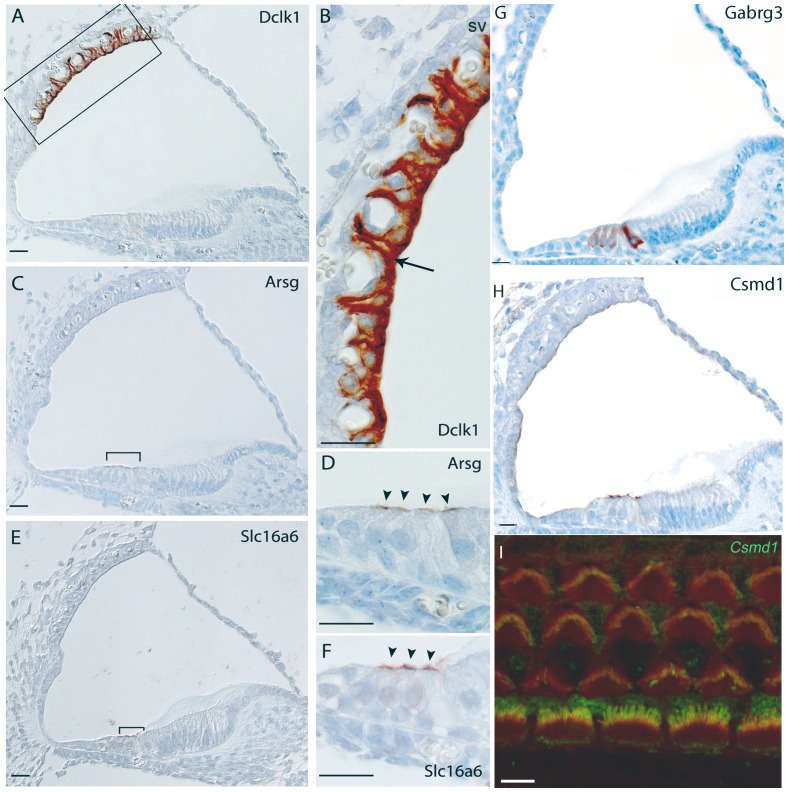
Immunohistochemistry in the mouse cochlea at P5 (P4 for Slc16a6 gene). Brown indicates positive staining. A, B) Expression of Dclk1, showing intense staining in the marginal cells including projections towards the basal cells of the stria vascularis (arrows in A,B); C, D) Expression of Arsg is localized at the top of sensory hair cells in the organ of Corti (bracket in C, arrowheads in D). E, F), Apical hair cells at P4, showing staining in outer hair cells of Slc16a6 of the organ of Corti (bracket in E, arrowheads in F). Note that these samples are from the C3HeB/FeJ strain, which is pigmented. Gabrg3 shows a striking specific expression in the outer and inner hair cells (arrowheads in H). In particular, the inner hair cells have the strongest staining. H, I) Expression of Csmd1 is localized at the top of sensory hair cells in the organ of Corti (arrowheads in H; I) confocal expression shows a strong staining localized in the stereocilia of inner hair cells and weak staining is also present in the stereocilia of outer hair cells. Csmd1 is labelled in green while rhodamine/phalloidin labels actin filaments of stereocilia in red. Merged image (yellow) indicates colocalization of Csmd1 with actin in stereocilia hair bundles. Scale bars: A–H: 20 µm; I: 5 µm.

Doublecortin-like kinase 1 (*Dclk1*) displays a strong pattern of expression in the marginal cells including projections towards the basal cells in the stria vascularis (Fig. 2 A,B). In the vestibular system, staining of *Dclk1* could be seen in the dark cells adjacent to the cristae (data not shown).

### 2) Expression in the Hair Cells of the Organ of Corti

Arylsulfatase G (*Arsg*) shows striking specific expression at the top of sensory hair cells in the organ of Corti (Fig. 2 C, D). No staining in the vestibular system has been detected.

Solute carrier family 16, member 6 (*Slc16a6*) shows expression at the top of the outer hair cells in the organ of Corti (Fig. 2 E, F), but weak staining is also present in the hair cells and supporting cells of the maculae and cristae of the vestibular system (data not shown). The staining of this gene is variable between mice of different genetic backgrounds: it is very clear in outer hair cells of C3HeB/FeJ mice (n = 4), while a more faint and less specific pattern of expression was detected in C57BL/6J-*Tyr^c-Brd^* mice (n = 4).

*Gabrg3* is a member of the GABA receptor gene family, a group of proteins involved in the GABAergic neurotransmission of the mammalian central nervous system. It shows a striking specific expression in the outer and inner hair cells. In particular, the inner hair cells have the strongest staining (Fig. 2 G). Heavy expression was also noted in the hair cells in the cristae of the vestibular system (data not shown).

*Csmd1*, CUB and Sushi multiple domains 1, expression was noted at the top of outer and inner hair cells in the organ of Corti (Fig. 2 H). To better understand the precise localization of this expression, a confocal microscopy analysis of whole mount preparations was also performed. The expression is strongly localized in the stereocilia of the inner hair cells but faint staining is also present in the stereocilia of the outer hair cells (Fig. 2 I). Strong expression was also noted in the hair cells in the maculae and cristae of the vestibular system (data not shown).

### 3) Expression in Multiple Cell Types in the Cochlea

**Figure 3:**
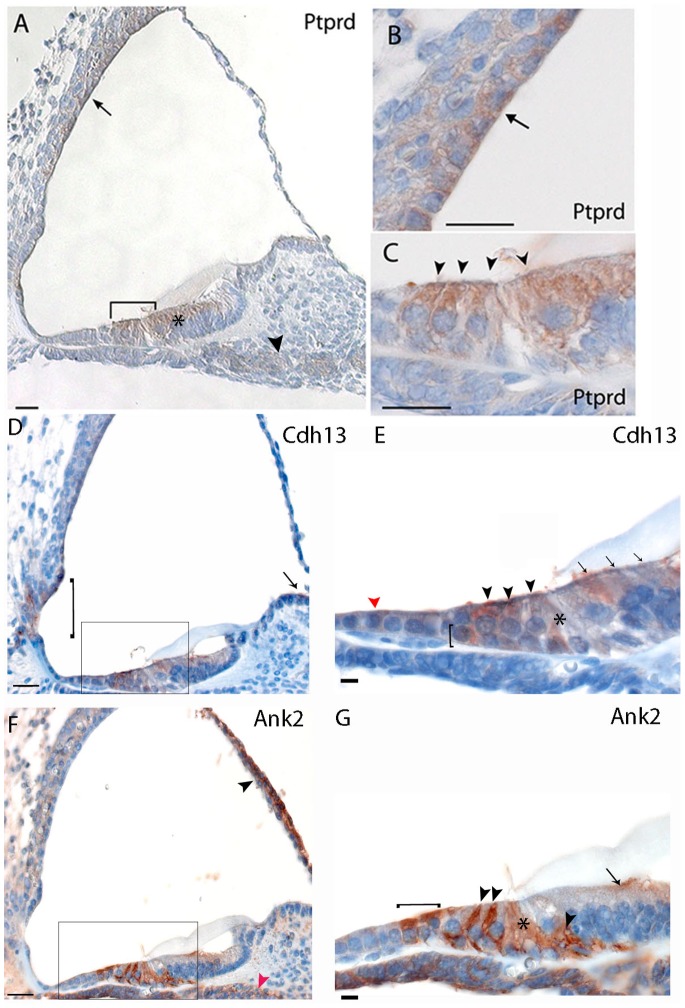
Immunohistochemistry in the mouse cochlea at P5. Brown indicates positive staining. A, B, C) Ptprd is localized in hair cells of the organ of Corti (bracket in A, arrowheads in C), in the marginal cells of the stria vascularis (arrow in A, B), in the supporting cells of the Kölliker’s organ (marked by an asterisk in A) and in the spiral ganglions neurons (arrowhead in A); D, E) Cdh13 is expressed in cells of Claudius (red arrowhead in E), outer and inner hair cells (arrowheads in E), Deiters’ cells (bracket in E) and pillar cells (asterisk in E), cells of the Kolliker’s organ (arrows in E) in the organ of Corti. Staining was also noted in interdental cells (arrow in D), stria vascularis (asterisk in D), spiral prominence and external sulcus cells (bracket in D). F, G) Ank2 could be noted in the Hensen’s cells (bracket in G), Deiters’ cells (arrowheads in G)and pillar cells (asterisk in G) in the organ of Corti. Moreover, Ank2 is expressed in the Reissner’s membrane (arrowhead in F) and cells of the Kolliker’s organ (arrow in G). Scale bars: A–C: 20 µm. D, F: 20 µm; E,G: 10 µm. Rectangles label the regions shown in higher magnification.

Protein tyrosine phosphatase, receptor type, D (*Ptprd*) is expressed in outer and inner hair cells, in the stria vascularis (more strongly in the marginal cells), in Kölliker’s organ and the spiral ganglion (Fig. 3 A, B, C), and in vestibular hair cells and supporting cells (data not shown).

*Cdh13,* a member of the cadherin superfamily, is mainly expressed in Claudius cells, outer and inner hair cells, Deiters’ cells and pillar cells, Kölliker’s organ and interdental cells; staining was also noted in the spiral prominence and external sulcus cells (Fig. 3 D, E).

Ankyrin 2 is a member of the ankyrin family, a group of molecules that link the integral membrane proteins to the underlying spectrin-actin cytoskeleton; *Ank2* expression was mainly noted in Hensen’s cells, Deiters’ cells, pillar cells and in Reissner’s membrane (Fig. 3 F, G).

**Figure 4:**
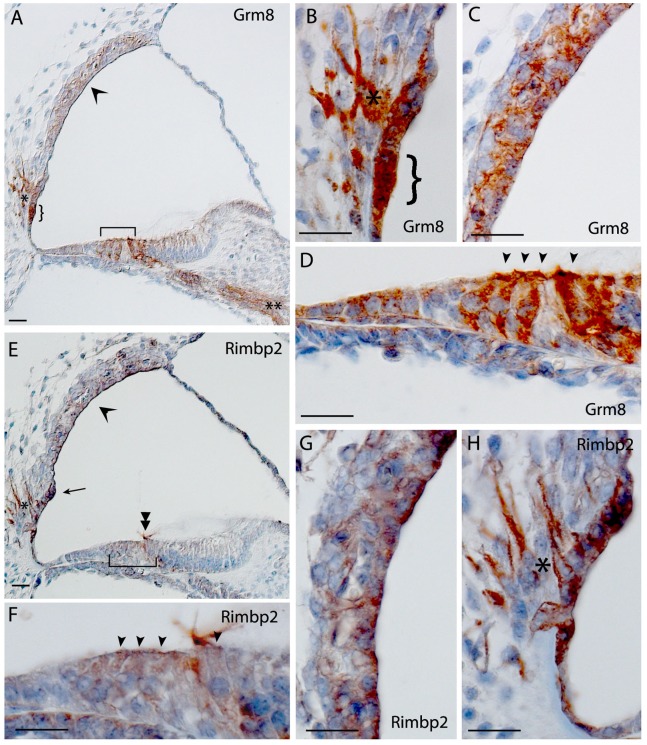
Immunohistochemistry in the mouse cochlea at P5. Brown indicates positive staining. A, B, C, D) Staining of Grm8 showing expression throughout the cochlea, most notably the root cells (curly brackets in A and B) and root cell processes (asterisks in A and B), the stria vascularis (open arrowhead in A, C), the hair cells (brackets in A, arrowheads in D) and also in the spiral ganglion of the neurons (double asterisks in A); E, F, G, H) Expression of Rimbp2 showing staining in hair cells (bracket in E, arrowheads in F), tectorial membrane (double arrowhead in E), the stria vascularis (open arrowhead in E, G) and in the spiral prominence (arrow in E) and root cell processes (asterisks in E and H). Scale bar indicates 20 µm.

Glutamate receptor, metabotropic 8 (*Grm8*) gave positive signals in the outer and inner hair cells, Claudius and Hensen’s cells, Kölliker’s organ and spiral ganglion neurons (Fig. 4 A, B, C, D). Staining of *Grm8* was also noted in the root cells, spiral prominence and stria vascularis, and there was strong expression in vestibular hair cells, supporting cells and neurons (data not shown).

RIMS binding protein 2 (*Rimbp2*) expression was noted in outer and inner hair cells, the lateral edge of the tectorial membrane, root cells, spiral prominence, spiral ganglion and in the marginal and intermediate cells of the stria vascularis (Fig. 4 E, F, G, H). Heavy expression was also noted in the vestibular hair cells, supporting cells and neurons (data not shown).

**Figure 5:**
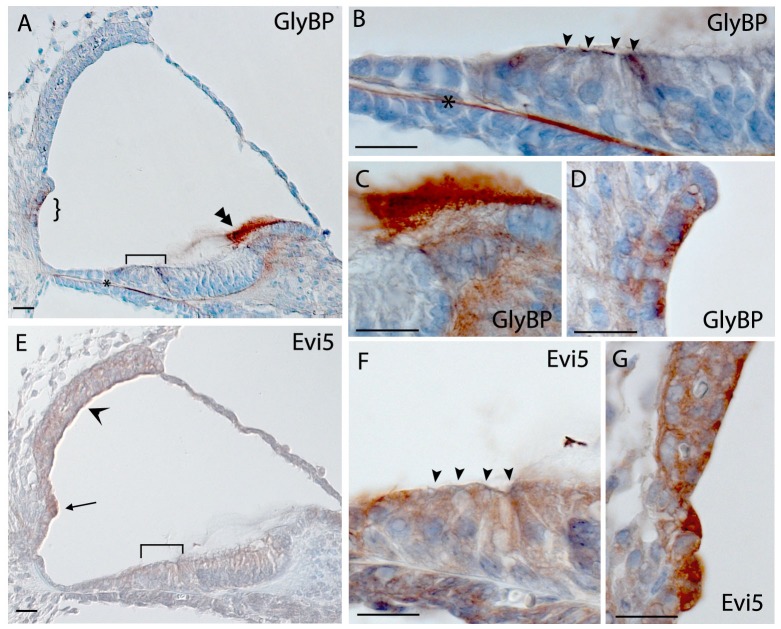
Immunohistochemistry in the mouse cochlea at P5. Brown indicates positive staining. A, B, C, D) Expression of GlyBP showing staining in hair cells (bracket in A, arrowheads in B), tectorial membrane (double arrowhead in A, C), root cells (curly brackets in A, D) and basilar membrane (asterisk in A and B). E, F, G) Staining of Evi5 localized throughout the cochlea, including hair cells (bracket in E, arrowheads in F) and spiral prominence and stria vascularis (arrow and open arrowhead in A respectively, G). Scale bar indicates 20 µm.

Centrosomal protein 104 kDa (*GlyBP/KIAA0562*) expression could be seen in the outer and inner hair cells and in Hensen’s cells; strong expression could also be seen in the spiral prominence and in the tectorial and basilar membranes (Fig. 5 A, B, C, D).

The expression of *Evi5* was noted in outer and inner hair cells, Deiters’ and pillar cells, cells of Kölliker’s organ, spiral ganglion, root cell processes, in the spiral prominence and in the stria vascularis, and weak staining could also be seen in Claudius and Hensen’s cells (Fig. 5 E, F, G). Strong staining was noted in hair cells, supporting cells and neural dendrites of the vestibular system (data not shown).

As regards the remaining genes, results were not conclusive, showing either no detectable labeling (*Amz2, Rpl5* and *Cmip*), or widespread labeling that appears non-specific (*Dffb, Fos*). Two different antibodies were tested for *Dffb, Amz2* and *Cmip*, but no specific pattern of labeling was seen with either. In addition, the RNA genes *Snora66* and *Snord21* were tested by *in situ* hybridisation using custom-designed Locked Nucleic Acid probes, but no labeling was seen in the ear.

These 12 genes expressed in specific patterns in the mouse cochlea were then included in a replication step by analyzing their genetic association using an independent cohort from the Silk Road, genetically and geographically very distant from the European population. A total of 9 genes (75%) were replicated; 4 genes were replicated at the SNP level (*CSMD1, ANK2, CDH13, DCLK1*) while an additional five (*ARSG, EVI5, GRM8, RIMBP2, SLC16A6*) were replicated at the gene level (see Table 4 and 5). In particular, the following SNPs were replicated: rs10091102-C (*CSMD1*, lowest p-value at PC1 = 0.022) and rs17045059-G (*ANK2*, lowest p-value at 4 kHz = 0.010) under an additive model; rs17195859-A (*CDH13*, lowest p-value at 6 kHz = 0.024) and rs9574464-G (*DCLK1,* lowest p-value = 0.008 at 0.25 kHz) under a dominant model. The strongest associations from the gene-based test were obtained for *GRM8* at 4 kHz (p-value = 0.006), and *EVI5* (p-value = 0.002) at 6 kHz.

**Table 4:** Results of the replication association study for the candidate SNPs of expressed genes.

Repl. model	add	add	dom	dom
HWE p-value	−	−	0.37	−
Conc	96.2	97.2	95.9	97.3
Info Score	0.98106	0.92767	−	0.95981
SNP type	imp	imp	gen	imp
Str	+	+	+	+
Trait	PC1	4 kHz	6 kHz	0.25 kHz
Maf	0.25	0.30	0.15	0.32
Repl. p-value	0.022	0.010	0.024	0.008
Repl. Beta±SE	0.04±0.02	0.08±0.03	0.07±0.03	−0.06±0.02
Meta- analysis Beta±SE*	0.08±0.03	0.25±0.06	0.27±0.06	−0.17±0.03
Eff. All	C	G	A	G
Top SNP	rs10091102	rs17045059	rs17195859	rs9574464
Gene	CSMD1	ANK2	CDH13	DCLK1

**Key**: Eff.All = effect allele; *from published work [3]; Repl. = replication; Maf = minor allele frequency; Str = strand; imp = imputed; gen = genotype; Info Score = imputation quality score form IMPUTE2 [6], Conc = concordance for the region encompassing the SNP (chunk dimension = 5 Mb); HWE = Hardy-Weinberg Equilibrium; add = additive; dom = dominant.

**Table 5:** Results of the gene-based association test for candidate expressed genes.

Gene	Total N. SNPs	N. Selected PCs	Variance Explained	P-value	Trait	Strand
RIMBP2	1228	60	90.652%	0.025	8 kHz	−
GRM8	1940	32	94.581%	0.006	4 kHz	−
ARSG	338	28	94.617%	0.033	1 kHz	+
SLC16A6	52	4	94.168%	0.039	PC2	−
EVI5	366	11	95.405%	0.002	6 kHz	−

**Key:** Total N. SNPs = total number of intragenic SNPs for each gene; N. Selected PCs = number of principal components explaining the largest amount of variance and used for the analysis (see Materials and Methods); Variance Explained = Total variance accounted for by the selected principal components.

**Figure 6:**
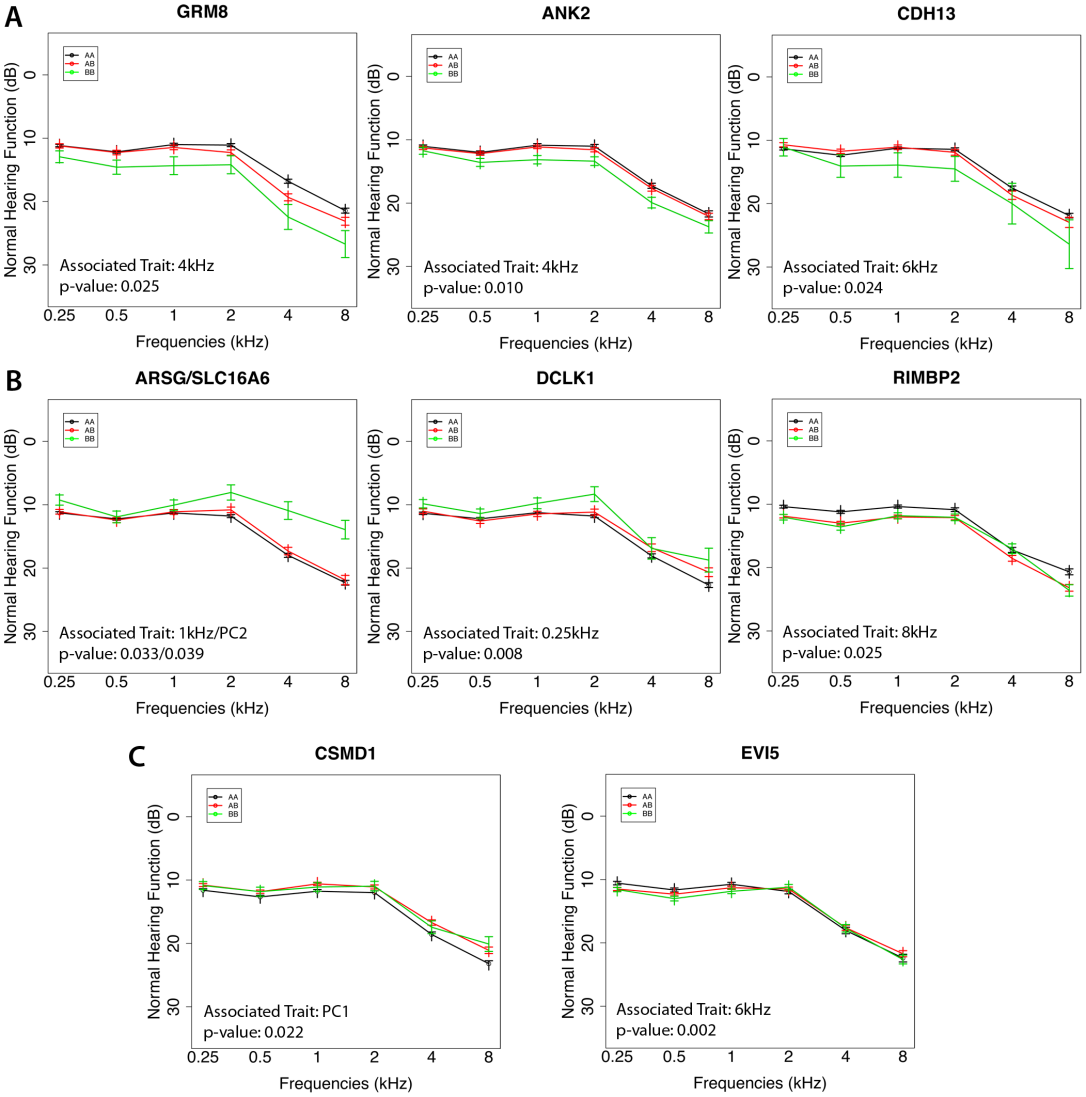
Genotype-phenotype relationship. A, B, C) The figure displays genotype-phenotype relationship for genes with evident differences among the three profiles. The x-axis represents frequencies (kHz), while the y-axis represents sex and age adjusted hearing thresholds (dB SL) with standard deviations, three different colors represent the corresponding genotypes (AA = black, AB = red, BB = green) and the number of subjects for each genotype is reported in brackets. A) *GRM8, ANK2* and *CDH13* show a deterioration of all the threshold levels for the homozygous BB. B) *SLC16A6*, *ARSG* and *DCLK1* display an improvement (ranging from 0.5 to 8 dB) at all frequencies for the homozygous BB. Similarly, *RIMBP2* presents an improvement for the homozygous genotype AA. C) *CSMD1* and *EVI5* do not display any particular pattern.

Finally the 9 genes with positive replication data were investigated to check for the possible presence of genotype/phenotype relationships by comparing the average audiometric curves. As shown in Fig. 6, seven genes (77%) (*GRM8, ANK2, CDH13, SLC16A6, ARSG, RIMBP2* and *DCLK1*) showed a distinct audiometric pattern for each genotype, with differences at some specific frequencies and intensities. *GRM8, ANK2* and *CDH13,* expressed in multiple cells type in the cochlea, showed a downward slope towards high frequencies for the homozygous BB individuals with a deterioration in all the thresholds compared with AA and AB genotypes (see Fig. 6A). Three other genes *SLC16A6*, *ARSG* and *DCLK1* with a specific expression pattern (in the hair cells in the organ of Corti or marginal cells of the stria vascularis) displayed a slope in which the homozygous BB subjects present an improvement in thresholds (ranging from 0.5 to 8 dB) at all frequencies, in particular for *SLC16A6* and *ARSG* genotypes. A slight improvement at all frequencies for the homozygous genotype AA was present for the *RIMBP2* gene (see Fig. 6B). As shown in Fig. 6C, *CSMD1* and *EVI5* did not show any specific pattern of genotype/phenotype relationship.

## Discussion

Despite recent progress, almost nothing is known about the molecular bases of variation of normal hearing, apart from genes identified as being directly involved in hereditary hearing loss and one gene (*GRM7*) recently described associated with age-related hearing loss (ARHL) in humans [13], [14]. Here, we report a strategy based on a) selection of genes from a previous GWAS [3], b) evaluation of their expression in the mouse, c) genetic replication of those showing a specific inner ear expression pattern in different and distant populations and, d) genotype-phenotype relationship of the replicated genes. This strategy proved to be an effective filtering and discovery approach, allowing us to uncover novel weakly-associated genetic variants. However, we cannot discard the remaining genes which could also be interesting candidates.

After the selection of 19 genes (see Materials and Methods) with suggestive GWAS p-values plus other criteria described above, expression studies demonstrated that 12 of them showed a clear expression pattern in the mouse cochlea. Nine of these were successfully replicated in an independent cohort of people geographically and genetically distant from those used to perform the first GWAS analysis [3]. This finding is extremely relevant taking into consideration that in four cases the replication was obtained using the same SNPs thus strongly suggesting that the genetic effect observed is of general relevance to multiple human ethnic groups [15]. In line with the criteria used by other large consortia [16], [17], we used a nominal significance of p<0.05 for replication using non-European populations.

Finally, the clinical step looking at genotype-phenotype relationships highlighted, in 7 cases, the presence of different audiometric profiles of the three genotypes. Two of them (*ARSG, SLC16A6*) showed striking expression at the top of sensory hair cells in the cochlea (*SLC16A6* only at the top of outer hair cells), *DCLK1* was expressed only in marginal cells of the stria vascularis, while the others (*CDH13, GRM8, ANK2* and *RIMBP2*) were expressed more widely in multiple cell types in the cochlea.

*DCLK1* is involved in several different cellular processes, including neuronal migration in the developing brain and in maturation of the nervous system [18]. Dclk1 expression within the cochlea is localized to the marginal cells of the stria vascularis, a structure that is essential for the secretion of K^+^ into the endolymph and for maintaining its associated endocochlear potential, a feature that enhances the electrochemical gradient across the top of hair cells, making them more sensitive. Dclk1 could be involved in the development of the extensive baso-lateral processes extended by the marginal cells. In this light, the hearing improvement present in homozygous BB subjects might be due to a variation in endolymph homeostasis.

*SLC16A6* and *ARSG,* which are located in the same locus overlapping each other and were both replicated under the additive model, display a downward slope for the homozygous BB genotype very similar to that shown by *DCLK1*. *SLC16A6* belongs to the solute carrier (Slc) family, whose members have already been associated with different forms of deafness [19], and *ARSG* is involved in hormone biosynthesis, modulation of cell signaling, and degradation of macromolecules. Both are expressed at the top of the hair cells, where the mechanical forces evoked by sound are translated into an electrical signal, so any variants in these genes could alter the sensitivity of hearing.

A genotype-phenotype difference, with a slight improvement of threshold in the homozygous AA subjects, is evident for *RIMBP2;* interestingly, it maps within the DFNA41 locus associated with dominantly-inherited progressive hearing loss [20]. *RIMBP2* is a member of a family of proteins that act as binding partners of the presynaptic active zone proteins RIMs [21] as well as for voltage-gated Ca^2+^-channels, such as CACNA1B and CACNA1D, the latter already known to be involved in deafness [22]. In this light, the strong staining of this protein in different cell types in the cochlea and the possible interaction with CACNA1D could indicate an important role in hearing function.

A similar audiometric slope for the three genotypes and in particular for the homozygous BB subjects is also evident for three genes expressed in multiple cell types in the cochlea: *GRM8, ANK2 and CDH13* (this last one also replicated under a dominant model, while the first two were replicated under a standard additive model). These three genes belong to families whose members have been already reported as expressed in the hearing system. *CDH13* belongs to the cadherin superfamily genes [23]. *ANK2* is a member of the Ankyrin family of proteins with protein domains found in TRP channels which may be important for the mechanosensitive channel responsible for hearing [24]. *GRM8* is a member, together with *GRM7*, of the group III metabotropic glutamate receptors (GRMs) which are neurotransmitter receptors [25]. Furthermore, GRM8 and GRM7 proteins show 87% homology and 76% identity to each other [26] and *GRM7* has been recently reported to be involved in ARHL [13], [14]. Consequently the strong expression of *Grm8* in the mouse cochlea supports a role for this gene.

As regards *CSMD1 and EVI5*, despite their strong expression in the inner ear and their replication in the Silk Road cohort (under the additive model), they didn’t show any specific genotype-phenotype audiometric pattern. Previous analysis of *Csmd1* mRNA expression by *in situ* hybridization and immunolabelling of neurons indicated that the primary sites of synthesis are the developing CNS and epithelial tissues [27], while we have shown that the staining is localized at the top of sensory hair cells, including in stereocilia. *EVI5* encodes a protein which binds to alpha and gamma tubulin, essential components of microtubules of the inner hair cells [28], [29].

As regards genes that were not replicated (*GABRG3, PTPRD* and *GlyBP),* their expression patterns suggest they are worthy of further investigation, and *GABRG3* and *PTPRD* both belong to families previously implicated in auditory function [30]–[32].

In conclusion, our approach confirms the usefulness of a multi-step method, combining several known techniques in order to further investigate the role of genes identified after a GWAS for hearing function, and increases our knowledge of the genes involved in normal hearing function that might also play a role in hearing loss including presbycusis.

## Web Resources

Hereditary Hearing Loss Homepage. Available: http://hereditaryhearingloss.org/Accessed 2013 Nov 5.

Hereditary Hearing Impairment in Mice. Available: http://hearingimpairment.jax.org/index.html Accessed 2013 Nov 5.

Mouse Genome Informatics. Available: http://www.informatics.jax.org/Accessed 2013 Nov 5.

Unigene. Available: http://www.ncbi.nlm.nih.gov/UniGene/ESTProfile Accessed 2013 Nov 5.

Minimac. Available: http://genome.sph.umich.edu/wiki/Minimac Accessed 2013 Nov 5.

Eurexpress. Available: http://www.eurexpress.org/ee/Accessed 2013 Nov 5.

Shield. Available: https://shield.hms.harvard.edu/Accessed 2013 Nov 5.

Morton Lab - Hearing Research Group. Available: http://brighamandwomens.org/Research/labs/BWH_Hearing/Cochlear_ESTs.asp Accessed 2013 Nov 5.

## Supporting Information

Figure S1 **Maps of cohorts investigated.** The figure shows the location of the cohorts used in the previous study and in the follow-up replication study.
(TIF)

Table S1 **Primary and secondary antibodies used in immunohistochemistry and confocal studies.**
(DOCX)
