# Correction: Expression and Replication Studies to Identify New Candidate Genes Involved in Normal Hearing Function

**DOI:** 10.1371/journal.pone.0091446

**Published:** 2014-03-07

**Authors:** The PLOS ONE Staff

Table 4 has been corrected for orientation and readability.

Please see the corrected Table 4 here:

**Table 4:** Results of the replication association study for the candidate SNPs of expressed genes.

Gene	Top SNP	Eff. All	Meta- analysis Beta±SE*	Repl. Beta±SE	Repl. p- value	Maf	Trait	Str	SNP type	Info Score	Conc	HWE p- value	Repl. Model
CSMD1	rs10091102	C	0.08 ± 0.03	0.04 ± 0.02	0.022	0.25	PC1	+	imp	0.98106	96.2	-	add
ANK2	rs17045059	G	0.25 ± 0.06	0.08 ± 0.03	0.010	0.30	4 kHz	+	imp	0.92767	97.2	-	add
CDH13	rs17195859	A	0.27 ± 0.06	0.07 ± 0.03	0.024	0.15	6 kHz	+	gen	-	95.9	0.37	dom
DCLK1	rs9574464	G	-0.17 ± 0.03	-0.06 ± 0.02	0.008	0.32	0.25 kHz	+	imp	0.95981	97.3	-	dom
