# Supplementary figures and images for: Expression and Replication Studies to Identify New Candidate Genes Involved in Normal Hearing Function
Source: PLoS One. 2014 Jan 14;9(1):e85352. doi: 10.1371/journal.pone.0085352 (PMC3891868; doi:10.1371/journal.pone.0085352)

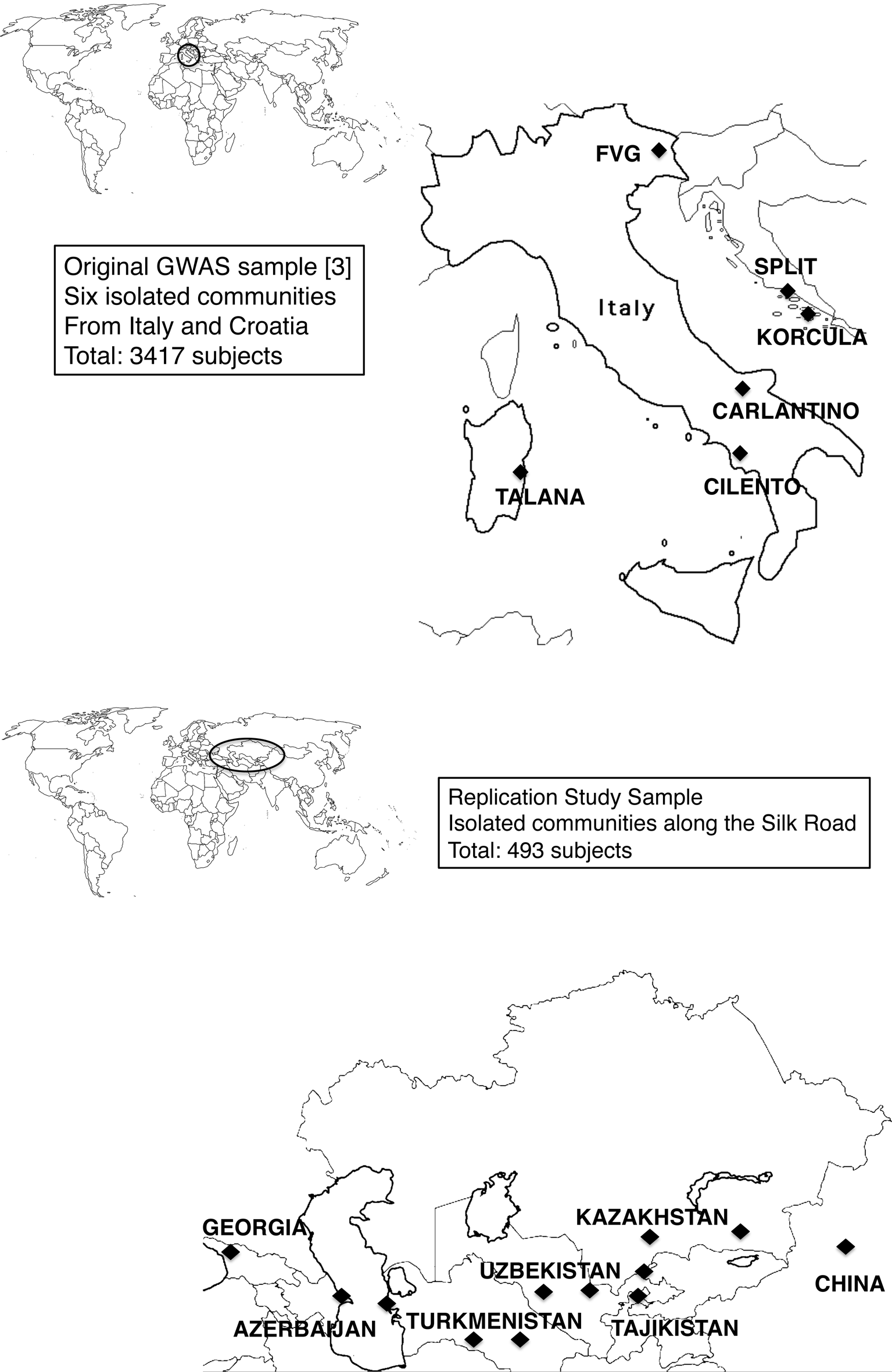

Supplement: Figure S1 — Maps of cohorts investigated. The figure shows the location of the cohorts used in the previous study and in the follow-up replication study. (TIF) [file pone.0085352.s001.tif]
