# Supplementary material for: Expression and Replication Studies to Identify New Candidate Genes Involved in Normal Hearing Function
Source: PLoS One. 2014 Jan 14;9(1):e85352. doi: 10.1371/journal.pone.0085352 (PMC3891868; doi:10.1371/journal.pone.0085352)
Supplement: Table S1 — Primary and secondary antibodies used in immunohistochemistry and confocal studies. (DOCX) [file pone.0085352.s002.docx]

Supplementary Table S1. Primary and seconday antibodies used in immunohistochemistry and confocal studies.

| **Antibody** | **Concentration** | **Company** |
| --- | --- | --- |
| anti-Dclk1 | 1:50 | Abnova, PAB2050 |
| anti-Arsg | 1:50 | Sigma Aldrich, HPA023245 |
| anti-Evi5 | 1:10 | Abgent, AP9168c |
| anti-Ptprd | 1:200 | Abcam, ab103013 |
| anti-Slc16a6 | 1:20 | Santa Cruz, sc-51325 |
| anti-GlyBP | 1:100 | Abcam, ab28773 |
| anti-Gabr3 | 1:50 | Santa Cruz, sc-7371 |
| anti-Cdh3 | 1:200 | Abcam, ab36905 |
| anti-Grm8 | 1:50 | Abcam, ab53094 |
| anti-Rimbp2 | 1:25 | Santa Cruz, sc-169182 |
| anti-Csmd1 | 1:50/1:200 | Santa Cruz, sc-68280 |
| anti-Ank2 | 1:50 | Santa Cruz, sc-28560 |
